# Contributions of park constructions to residents’ demands of ecosystem services consumption: A case study of urban public parks in Beijing

**DOI:** 10.1371/journal.pone.0259661

**Published:** 2021-12-15

**Authors:** Sibo Wang, Tingwei Li, Dongdong Li, Hong Cheng

**Affiliations:** 1 Research Institute for Eco-civilization CASS, Beijing, 100028, China; 2 Department of data research, Beijing E-Hualu Information Technology Co., Ltd., Beijing, 100430, China; 3 Department of Marxism, North China University of Technology, Beijing, 100144, China; Northeastern University (Shenyang China), CHINA

## Abstract

Urban public parks can provide convenience for residents to get close to nature and provide places for daily ecosystem services. It is of practical and theoretical significance to choose urban public parks as the entry point to explore the changing trends and supply paths of urban residents’ daily ecosystem service consumption. Based on the government ‘s research? of urban public parks in Beijing from 1993 to 2018, this study explores the residents’ ecosystem services consumption demands and the contributions of park constructions to these demands. The results show that: (1) in the past 25 years, the frequency, duration, participation rate, and evaluation of people’s daily ecosystem service consumption have increased significantly. In other words, the ecosystem services demands are increasing. (2) different constructions of a park have distinct contributions to the increasing demands of ecosystem service consumption. The contributions from constructions of the natural landscape and the infrastructure have been in decline since 1993, yet they contribute the most to the demands of residents’ ecosystem services consumption until 2018. The contributions made by constructions of management and maintenance, and transportation around urban public parks have been on the rise and the significant points occurring after the 2008 Olympic Games. Our research proposes a method to determine the relation between the demands of residents’ ecosystem services consumption and the contributions of park constructions to these demands, which has significant implications for optimizing the constructions of urban public parks to better meet the demands of ecosystem services consumption.

## 1. Introduction

The world is experiencing an unprecedented urbanization trend that 60% of the land will be urban in 2030 [1]. China’s urbanization rate, for instance, has risen rapidly from 17.92% to 60.60% in 1978–2019. In the process of urbanization, many natural ecosystems have been replaced by artificial systems, such as factories, which led to a prominent contradiction between the supply and demands of ecosystem services. Therefore, exploring the daily ecosystem service consumption trends and supply mode of urban residents has important practical and theoretical significance.

Urban public parks serve as hubs for entertainment, education, disaster prevention and mitigation functions and specially for high-quality ecosystem services that are accessible to and highly valued by visitors. Therefore, urban public parks can be a starting point to study the trend of ecosystem service demands and how urban park construction can meet residents’ daily demands in consuming ecosystem services.

The related literature of urban public parks is rich and can be organized according to research methods and perspectives: (1) In terms of research methods, research in urban public parks has evolved from independent research on ecology or sociology to integration research. The first branch of studies explored the relationship between the constructions of parks and ecosystem service capabilities based on ecological methods. Agnes et al. revealed potential of single trees in urban environment for the small-scale climate regulation. They selected five healthy, mature trees free of the disturbing (additional shading) effects present in any other trees or artificial objects. Results showed that the five investigated trees have a significantly greater impact on the components of the radiation budget, while their modification of air temperature and humidity is less obvious [2]. Vanwalleghem et al. used regression kriging (RK) to analyze the degree to which physiographic versus ecological variables influence spatial-temporal variation in understory microclimate conditions [3]. The second branch of research looking at the constructions of urban parks based on sociological methods [4,5] has led to significant findings. It is believed that the constructions including landscape, spatial distribution, management, and infrastructure of parks will affect the consumption [4–6], loyalty and satisfaction of visitors [7–10]. In terms of park management, the manager compensates for the management and maintenance costs of parks not by raising prices, but by taxation. If the ticket prices are raised, the total social welfare and equity will be affected [11]. Free opening is the primary mode of urban public parks management and maintenance in future [12]. The spatial distribution of urban parks will affect the function efficiency together with human well-being and fairness perception [13–15]. The urban public park should expand the scale and extend the opening hours to improve the service capacity and selection diversity of parks at the temporal and spatial level [16]. Some scholars have conducted in-depth research through questionnaires and data crawled from the Internet to find the motivation of residents’ demands in consuming urban public parks. Looking at behaviors of visitors in urban public parks, Kamri found that visitors visit a national park (Bako National Park) for the four factors. Specifically, challenge excursion, social trip, nature tour and getaway outing [5]. Investigations on behaviors of visitors in 13 public green spaces in Sheffield, UK indicated that visitors are most concerned about the ecological environment and high-quality scenery provided by urban public parks [17]. Another research investigated high-frequency words based on comments of several parks (Beijing Olympic Park, Nanhaizi Park, Tongzhou Grand Canal Park, Dongxiaokou Forest Park, and Mangshan National Forest Park), showing that enjoying the ecological environment, natural landscape, fresh air, and other ecosystem services are the major motivations of visiting urban public parks. Besides, by providing free places for family traveling and gatherings, urban public parks are transformed into public welfare undertakings to improve people’s well-being [18] as well as improve the physical and mental health of the locals in terms of its benefits [19], which caters to the strong demand for parks of the retired elderly in aging societies such as China [20–23]. The third branch of studies combined ecological and sociological methods to explore the constructions of urban parks and their contributions to the public ecosystem demands. B.M. researched the boundaries and interaction mechanisms of ecosystems and social systems from the perspective of landscape planning to study the ecosystems and society system integration development path [24]. Lee explored the impact of urban public parks on the sustainable development of cities from the perspective of tourism, and proposed that urban public parks are important places for residents to consume daily ecosystem services and can promote the sustainable development of cities [25]. Teja Tscharntke and Peter H Verburg discussed the supply and demand of urban park ecosystem services based on the perspectives of biodiversity and sustainable land use [26,27].

(2) The research perspectives of ecosystem services have undergone a shift from functions and value evaluation to supply capabilities. Some studies have researched the service function and value evaluation of urban public parks Paul, et al. carried out functional research and value measurement of urban parks in their own countries. Jun Yang explored the spatial and temporal changes of parks to examine the dynamic impact of parks on urban ecosystem services through research on urban fringe areas. With the maturity of research and measurement methods, the subject of research has gradually shifted to the supply capabilities ecosystem service [28–31]. Heather et al. sought to determine whether specific sectors of the urban population have access to green space in close proximity of their homes and localized patterns of use in (municipal) urban parks and neighborhood parks. This research examined preferences, perceptions, and access barriers of parks and urban green space using on-site individual semi-structured interviews and systematic observations of behavior among a purposive sample of urban residents [32]. Norris et al. found that old-growth woodlands are shown to attenuate surface temperature more effectively than native species plantations, suggesting a greater efficiency of energy degradation in complex forest ecosystems, particularly at higher temperatures [33]. Liang et al. found that urban greening, especially urban forests, significant resists the effect of urbanization on annual variation in negative air ion concentrations (NAIC) [34]. It has also been suggested that ecologically, tree species serve to transform heavy metals [35,36]. Du confirmed that vegetation has different capacities in humidification, cooling, anti-pollution, and noise reduction in Xiangshan Park. Cotinus coggygria mixed forest produces the largest ecological effect, while the lawn the smallest [37]. Sodoudi et al. selected 25 green areas with five different spatial configurations and five vegetation types and revealed that the spatial configuration of a green area can strongly influence its cooling effect [38].

To sum up, high-quality ecosystem services are the major motivation for residents to visit urban public parks. The existing research on urban public parks focuses on social economics and ecology and some questions are left unexplored. First, previous research mostly centers on the factors that affect parks usage, not how much these factors have contributed to residents’ demands for ecosystem demands. Second, the object of existing literature includes only the impact of some constructions, such as the natural landscape, and internal constructions, such as infrastructure. Little literature focuses on other impacts of constructions such as management and maintenance, and surrounding public transportation, which cannot be ignored when analyzing the contributions of constructions to residents’ demands in park consumption. Third, the existing studies only explore the contribution of a single factor such as landscape construction. Few scholars conduct systematic research on the contributions of multiple construction factors to residents’ ecosystem services demands.

This paper concentrates on the changing trends of demands for residents’ ecosystem services consumption and contributions of park constructions to these demands based on government-led surveys and questionnaires. Specifically, the study used a production function and a panel model to strip off the contributions of urban public park constructions to residents’ ecosystem demands, and to explore the general rules supporting urban ecosystem planning and designing.

## 2. Materials and methods

### 2.1. Data source

Residents’ daily visits to urban public parks were mainly measured by time, and the daily visiting time of urban residents is obviously affected by regional climate differences. The more the survey sites are, the more intrusive these factors will interfere with the results, and the more difficult to control the results. The survey sites are selected with caution in order to control for selectivity differences, regional climate, and other interference factors on the daily urban public park utilization. We choose Beijing is selected as the research object given its representativeness among Chinese cities.

According to the above explanation, combined with the purpose of the study, the data required for the study consists of government-led surveys and questionnaires.

1. Government surveys. The data used were derived from the Beijing Garden Yearbook, Beijing Garden and Greening Yearbook, and Beijing Park Year-book from 1993 to 2018. Part of the data came from the Beijing Municipal Bureau of Land-scaping and Park Management and Maintenance. Chaoyang District, Fengtai District, Shi-jingshan District, Haidian District, Mentougou District, Fangshan District, Tongzhou District, Shunyi District, Changping District, Huairou District, Pinggu District, and Yanqing District were finally selected and a total of 12 urban public parks in each district were used as re-search objects
2. Questionnaires. To investigate the trend of residents’ daily demands for ecosystem service consumption, we randomly sampled visitors in two parks and a total of 361 questionnaires were obtained. Questionnaires were collected from 12 urban public parks in Chaoyang District, Fengtai District, Shijingshan District, Haidian District, Mentougou District, Fangshan District, Tongzhou District, Shunyi District, Changping District, Huairou District, Pinggu District, and Yanqing District. 361 questionnaires were completed by 210 males and 151 females, with an average age of 48.7. History data came from respondents’ memories.

### 2.2. Model description

According to the discussion above, the analysis of the constructions contributions to demands of residents’ ecosystem services consumption mainly considers the following four aspects: natural landscape construction, infrastructure construction, management and maintenance construction, and surrounding public transportation construction.

Natural landscape is the groundwork for an urban public park. The main purpose of residents to visit park daily is entertainment as well as to improve their physical and mental health. Parks with diverse natural ecological environment quality are more attractive. The per capita area and diversity of natural landscapes are directly associated with the phenomenon of crowding and viewing of urban public parks, which have a greater impact on park consumption demands and satisfaction. If the per capita area is too small, urban public parks are more likely to be crowded and the user experience will deteriorate. The spatial distribution of urban public parks that determines the convenience and accessibility of residents’ daily visits to the park has a greater impact on residents’ park consumption demands.

Infrastructures in urban public parks provide convenience for visiting, enrich options forentertainment, and improve other services of urban public parks. Footpaths and fitness facilities are important parts of the infrastructure to meet residents’ various daily demands for fitness. Besides, the construction of convenient facilities such as road chairs (stools) and toilets response to their resting and physiological needs. Therefore, improvement in the number, types, maintenance of footpaths and facilities will increase the residents’ daily demands for ecosystem services consumption.

The main construction of an urban public park is the management and maintenance when the park is put into formal operation. Construction of management and maintenance is an important guarantee for parks to provide residents with sustainable services. Management and maintenance can upgrade or maintain the natural landscape and infrastructures, organize diverse amusement projects, and manage the opening time. The improvement of the personnel professions is the key to improve the management and maintenance. More parks without walls or longer opening times of parks facilitate residents’ entry to the park. These management and maintenance constructions are all conducive to the increase in residents’ demands for ecosystem services.

The construction of public transportation around the park provides convenience for visitors who live far away to enter the park. Easy transportation shortens the time for visitors to enter the parks, prolong the experience time of residents in the parks, extends the daily effective service radius of the parks and make it possible for residents to visit more frequently.

This study used the Cobb-Douglas production function *Y* = *AK^α^L^β^* to analyze the constructions and their contributions to the demands of residents for consuming ecosystem services using the data of urban public parks in Beijing. Eq 1 is the models we modified, while Eq 2 takes the logarithm of both sides and expands the model.

$$Y_{\left(it\right)}=AX_{k_{it}}^{a_{k}}$$ (1)

$$\ln\;Y_{\left(it\right)}=\alpha_{0}+\sum_{k=1}^{n}\alpha_{k}\ln X_{k_{it}}+\varepsilon$$ (2)

where, *i* and *t* represent the targeted district and the year, respectively. *Y* represents the residents’ demands for daily park consumption, *X* represents the contributions made by constructions to the residents’ park consumption demands, ε represents the random error, and the elastic coefficient *α_k_* represents the flexibility of factor *X_k_* to meet the residents’ demands for visiting urban public parks.

## 3. Analysis and results

### 3.1. Statistical description analysis

#### 3.1.1. Analysis based on government survey data

We found that the number of urban public parks in Beijing providing free services to residents exceeds 90%. It is impossible to measure the residents’ demands for ecosystem services based on the actual expenditures of residents. Therefore, this study used the average stay time in parks per resident to measure residents’ demands for park consumption in each sampled area. The calculation of demands of a given park consumption is shown in Eq 3.


$$demands=\frac{annual\;vistors}{permanent\;population\times average\;stay\;time\times 365}$$ (3)

The consumption frequency, duration, and participation of daily ecosystem services can be described by frequency and duration, and participation of urban public parks, as shown in Table 1. With the development of society, the accelerated constructions of urban public parks and changes in the design concepts and operation methods of urban parks, more people are choosing parks for recreation. The annual visitors, the average stay time per visitor, and the average stay time per resident (demands) in the 12 sample areas in Beijing have risen from 23,761,100 persons, 25.42 minutes, and 0.39 minutes in 1993 to 62,205.33 person, 89.1 minutes, and 9.69 minutes in 2018. The survey also shows that the average number of visitors in urban public parks per week has increased from 0.59 in1994 to 1.51 in 2019. Based on the number of visitors in the 12 sample districts, the actual number of park visitors rose from 774,500 to the current 7,922,200. Considering the permanent population of the 12 sample areas, the participation rate of residents has increased significantly, from 19% (1994) to the 51% (2019). The increasing numbers indicate that the demands of ecosystem services consumption have increased significantly over the years.

**Table 1:** Changes of daily demands of ecosystem services consumption in Beijing.

Year	Consumption frequency and duration of ecosystem services			Participation of daily ecosystem service		
	Annual visitors	Average stay time per visitor	Average stay time per resident	Average number of visits (times / week)	Actual number of visitors (10 thousand)	Participation rate of residents
25 years ago	2376.11	5.69	25.42	0.59	77.45	19
20 years ago	3554.47	7.78	30.21	0.66	103.57	23
15 years ago	4649.53	8.8	38.45	0.73	122.49	23
10 years ago	11203.09	16.64	49.12	1.02	211.22	31
5 years ago	25389.5	26.53	65.21	1.14	428.3	45
2019	62205.33	39.69	89.10	1.51	792.22	51

#### 3.1.2. Analysis based on questionnaires

As shown in Table 2, residents for the purpose of experiencing commercial entertainment projects and enjoying free entertainment facilities have dropped from 71 (1994) to the current 62 (2019). The corresponding proportions were reduced from 32.87% and 28.70% to 9.38% and 18.75% respectively. Instead, residents for the purpose of viewing animals and plants and enjoying ecological environment have risen from 34 and 46 (1994) to 61 and 97 (2019), and the corresponding proportions have increased from 15.74% and 21.30% to 27.23% and 43.30%. In summary, experiencing commercial entertainment projects was the main purpose of people visiting the park 25 years ago and enjoying high-quality ecosystem services is the main purpose nowadays.

**Table 2:** Numbers of Beijing residents visiting urban public parks.

Year	Commercial entertainment	Free entertainment facilities	Animals and plants	High-quality ecosystem services	*Others
25 years ago	71	62	34	46	3
20 years ago	61	65	36	51	4
15 years ago	56	62	45	59	2
10 years ago	41	55	48	74	6
5 years ago	36	47	59	80	2
2019	21	42	61	97	3

*: Including visits organized by companies, visits without specific purpose, etc.

**Source**: Questionnaires of park visitors.

The questionnaires describe the changes in the residents’ evaluation of urban parks in Beijing, as shown in Table 3. Although urban public parks are sometimes too crowded to use, with the construction of parks in Beijing, the use of urban public parks has increased year by year according to park staff and the public. The score of ecological landscapes has risen from 60.2 (1994) to 95.1 (2019). The quality of ecological landscapes has been greatly improved, which also suggests an increase in the satisfaction of Beijing residents with consuming daily ecosystem service consumption increased. Score of urban public parks utilization has increased from 56.1 points (1994) to 89.7 points (2019). The scale and quality of urban public parks have both improved, which indicates that urban public parks congestion has been eased and residents’ daily consumption of ecosystem services is guaranteed.

**Table 3:** Evaluation changes of Beijing residents’ daily ecosystem services consumption.

Year	Ecological landscapes (1–100)	Congestion (1–100)
25 years ago	60.2	56.1
20 years ago	65.1	60.9
15 years ago	72.3	63.1
10 years ago	79.8	72.1
5 years ago	87.4	80.2
2019	95.1	89.7

### 3.2. Empirical analysis

#### 3.2.1. Models construction

According to the literature review, this study uses the average daily travel time per person in the sample area to measure the demands for urban public park consumption. The dependent variables are the annual number of tourists, the average stay time per visitor and the average stay time per resident in the urban public parks. The independent variables are classified into 4 categories of 11 variables, including natural landscape construction, infrastructure construction, management and maintenance construction and public transportation construction. The proportion of people over 60 years old is used as the control variable, as shown in Table 4.

**Table 4:** Descriptions of potential factors affecting residents’ park consumption demands.

Type	Variables	Definition	Unit	Average value in 1993	Average value in 2018
Consumption demand	Residents’ Park consumption demands	The average daily use of parks.	Minutes	0.39	9.69
Natural landscape construction	Per capita area	Average urban public park area per person.	HA / person	6.93	16.3
	Spatial distribution	Service coverage rate of 500m radius.	%	50.52	80
	Landscape diversity	Proportion of grassland and water area in total area.	%	4.81	8.35
Infrastructure construction	Footpaths	Length of footpath per 100 hectares.	km / 100 ha	0.06	0.46
	Fitness facilities	Number of fitness facilities per 100 hectares.	Pieces/ha	1.62	2.46
	Public chairs (stools)	Number of street chairs (stools) per 100 hectares.	Pieces/ha	5.93	13.02
	Toilets	Number of toilets per 100 hectares of park green space.	Sets/ha	0.34	0.71
Management and maintenance construction	Average opening time	Average daily opening hours of parks.	Hours / day	15.67	21.13
	Parks without walls	Proportion of areas in the parks without walls.	%	16.70	64.12
	Personnel with professional qualifications	Proportion of maintenance personnel holding qualifications of (civil) engineering and maintenance.	%	15.67	50.34
Public transportation construction	Visitors taking public transportation	Proportion of people who choose public transport mode to enter the park.	%	5.2	35.5
Control variables	People over 60 years old	Proportion of permanent residents over 60 years old in each district	%	12.13	16.82

**Note:** ****p< 0.01, *** p< 0.05, ** p< 0.1and * p< 0.15.

Based on government survey data, empirical analysis aims to find the contributions of constructions to residents’ demands for ecosystem service consumption in 12 districts of Beijing in the past 26 years. Tests of collinearity and robustness are required before empirical analysis. Stata 14.0 was used to do the analysis. The collinearity test mainly used the method of variance inflation factor (VIF). As shown in Table 5, independent variables of VIF are all less than 2.5 which confirms that no serious multi collinearity is detected in the dataset.

**Table 5:** Results of collinearity test (VIF) and robustness test.

Variables	Collinearity test		Robustness test			
	VIF	1/VIF	coefficient	*t-*value	coefficient	*t-*value
Residents’ Park consumption demands	1.34	0.75	-	-	-	-
Per capita area	1.52	0.66	0.345^a^	5.24	1.671^b^	2.8
Spatial distribution	1.67	0.6	0.102^b^	2.43	0.100^b^	2.41
Landscape diversity	1.23	0.81	0.089^c^	1.7	0.092^c^	1.67
Footpaths	1.46	0.68	0.004^c^	1.82	0.005^c^	1.84
Fitness facilities	1.78	0.56	0.009^c^	1.7	0.010^c^	1.73
Public chairs (stools)	2.12	0.47	0.004^c^	1.66	0.006^c^	1.67
Toilets	2.42	0.41	0.002^d^	1.53	0.002^d^	1.56
Average opening time	1.67	0.6	0.19^b^	2.51	0.21^b^	2.49
Parks without walls	1.54	0.65	0.004^c^	1.74	0.007^c^	1.79
Personnel with professional qualifications	1.46	0.68	0.008^a^	2.9	0.009^a^	3.09
Visitors taking public transportation	1.78	0.56	0.009^c^	1.65	0.009^c^	1.69
People over 60 years old	2.01	0.5	0.178^c^	1.71	0.167^b^	1.69
Mean	1.69					

**Note:**

a: p< 0.01

b: p< 0.05

c: p< 0.1and

d: p< 0.15.

To test the robustness of the model, the ratio of the area of urban public parks to the urban area was used to replace the variable of the per capita area, and the parameters of the measurement model were re-estimated. The sign of regression coefficients of the independent variable did not change. The p-values of area ratio and the per capita area were 0.017 and 0.00, respectively, and t-values were 2.80 and 5.24, respectively, both of which changed little. Besides, the variable footpaths (per 10,000 people have) were used to replace the variable footpaths (per 100 hectares). There were no significant changes in the coefficients, and t and p values significance. The coefficients and significance of other indicators were relatively stable and model was robust

As shown in Table 6, the fixed effects regression panel model was finally selected by the examination of F, BP-LM and Hausman. The results showed that auto-correlation was present in two sets of panel data and heteroscedasticity in some data. To avoid the influence of heteroscedasticity, the fixed-effect models used in the analysis were estimated to correct the auto-correlation and reduce heteroscedasticity problems.

**Table 6:** Tests for model selection.

Test method	Purpose	Result	Model
F-Test	To compare FE and Pooled model	Prob > F = 0.0002	FE > Pooled
BP-LM Test	To compare RE and Pooled model	Prob > chi2 = 1.0000	Pooled > RE
Hausman Test	To compare FE and RE model	Prob>chi2 = 0.0001	FE > RE

**Note**: FE means fixed effect; Re means random effect.

Three models were constructed based on the fixed effect model. The first two models feature a panel econometric analysis based on the overall data to analyze the contribution of park construction to park demand in the recent 30 years. The last model divides the overall data into five stages, and constructs five panel econometric analysis models for each period, so as to calculate the contribution of park construction to park demand in different periods. The first model is based on the whole sample and the panel data contains 12 panel numbers and 26 periods. The second model slices 25 years (1993–2018) into 5 smaller periods to re-examine the contribution of the different constructions of urban public parks to residents’ ecosystem demands, and the panel data contains 5 periods and 60 (12×5) numbers. The separation eliminates the possible cyclical fluctuation deviations in each period and unearths the characteristics of the data. The stock data is the mean of the head and the tail stock data, and the flow data is the mean of all data. We found that the measurement results of the model with 5 periods were roughly the same as the measurement results of the model with 26 periods, but the fit ability of the former model was better than the latter.

The third model was to find the trend of contributions of each kind of construction with time going by. The whole sample (1993–2018) was subdivided into 5 smaller samples analyzed in a panel model. Compared to the second model, the third model consists of 5 models. Every model contains 5(or 6) periods and 12 numbers.

#### 3.2.2. Contributions of constructions to residents’ park consumption demands (model 1 & 2)

**Fig 1:**
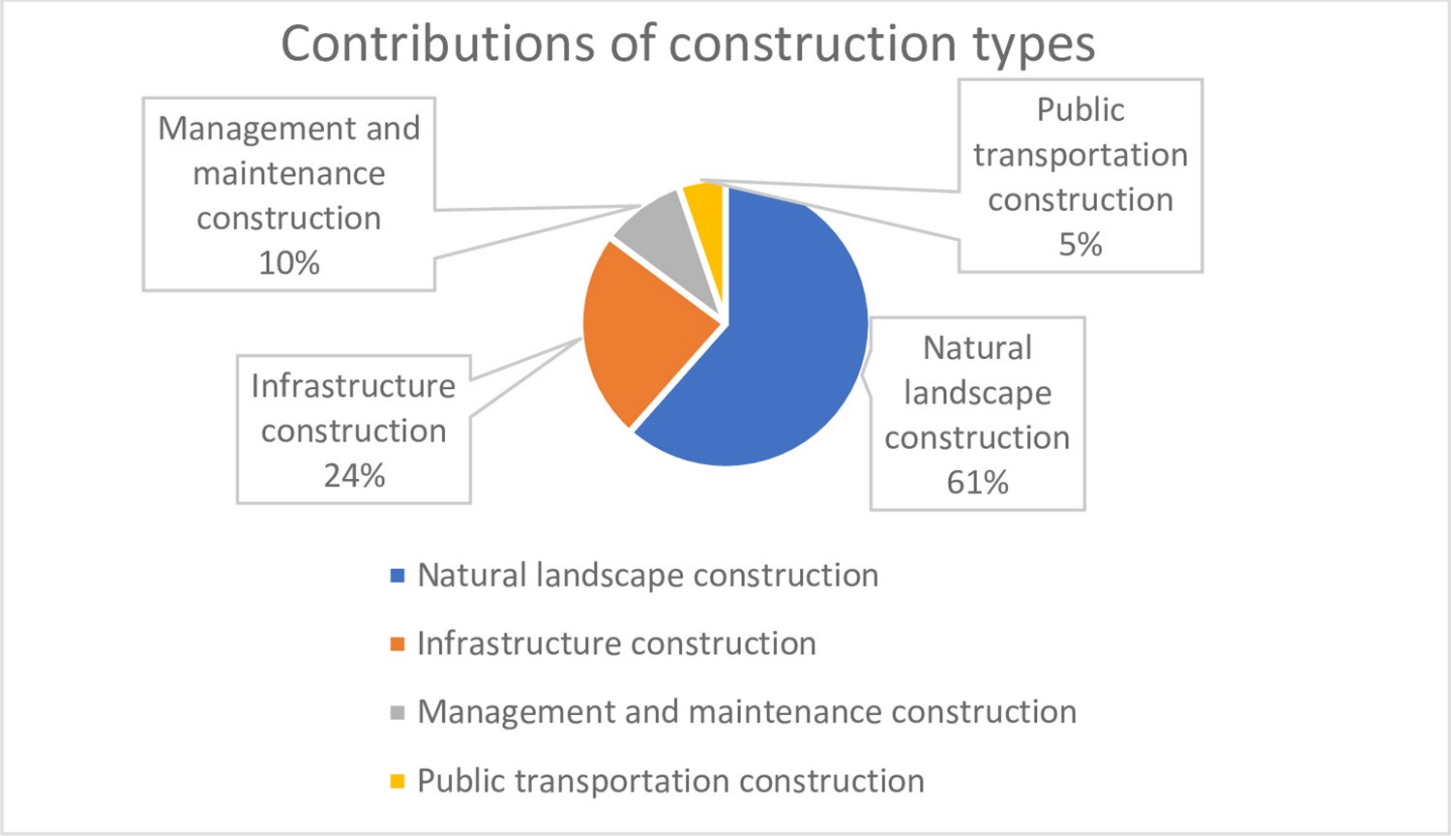
Contributions of construction types.

**Fig 2:**
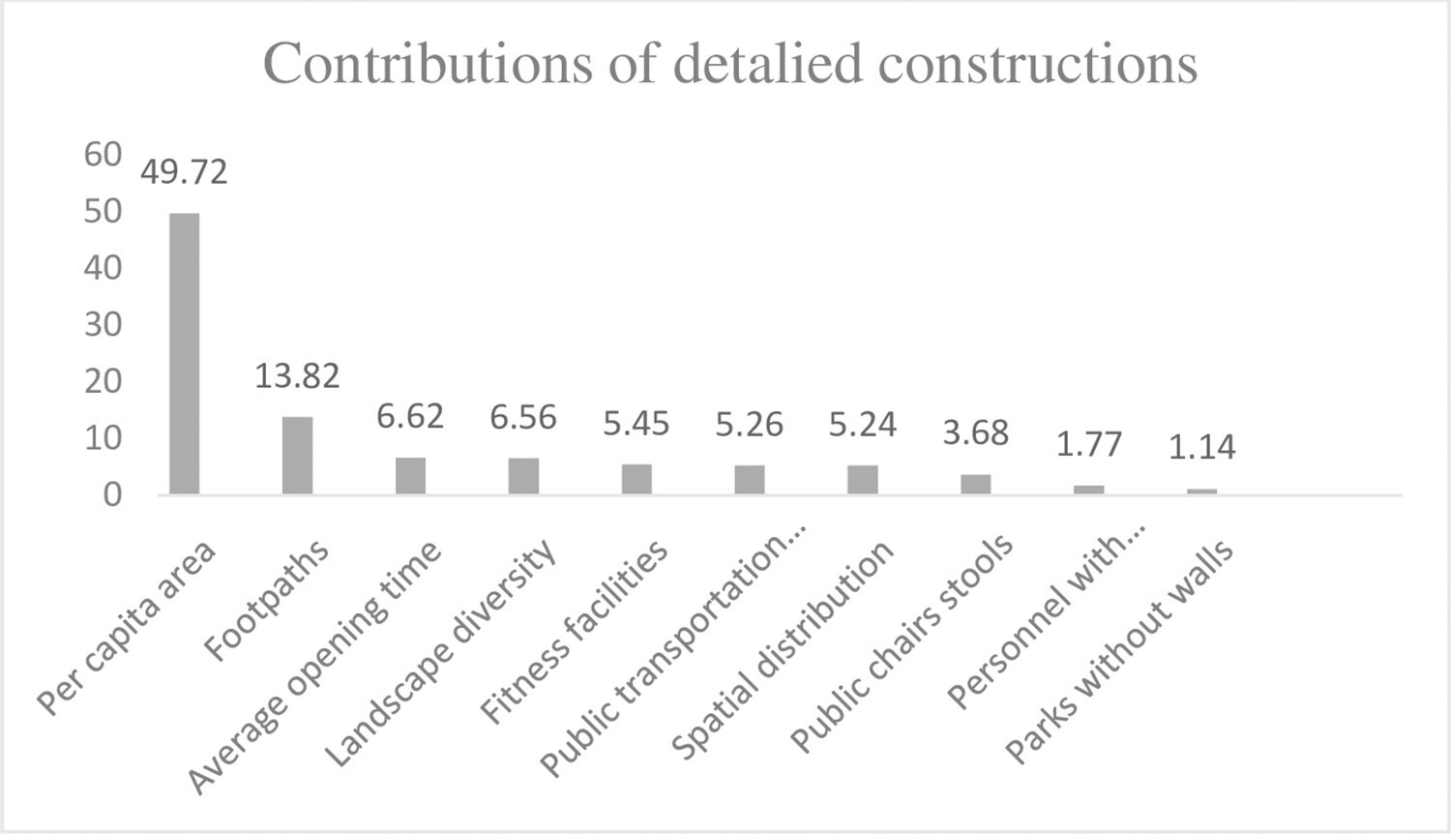
Contributions of detailed constructions.

The results of the former two models are shown in Tables 7 and 8. The contributions of four construction types to the residents’ demands for ecosystem service consumption are in a descending order (%) as shown in Fig 1. The specific contribution rates are shown in the Fig 2 where ‘per capita area’ makes the largest contributions and accounts for half of the total contribution.

**Table 7:** Statistics of contributions of different constructions to residents’ park consumption demands (26 periods).

Type	Variables	Annual growth (%)	Elastic coefficient (%)	*p*-Value	Contribution (%)	Whole contribution (%)
Natural landscape construction	Per capita area	5.75	0.351	a	40.77	56.25
	Spatial distribution	2.05	0.122	b	8.82	
	Landscape diversity	2.94	0.091	c	9.44	
Infrastructure construction	Footpaths	137.82	0.002	c	9.72	23.20
	Fitness facilities	24.15	0.012	c	10.22	
	Public chairs (stools)	36.67	0.003	c	3.88	
	Toilets	14.79	0.001	d	0.52	
Management and maintenance construction	Average opening time	1.39	0.22	b	10.79	14.27
	Parks without walls	11.36	0.005	c	2.00	
	Personnel with professional qualifications	8.85	0.007	a	2.19	
Public transportation construction	Visitors taking public transportation	23.31	0.008	c	6.27	6.27

**Note:**

a: p< 0.01

b: p< 0.05

c: p< 0.1and

d: p< 0.15.

**Table 8:** Statistics of contributions of different constructions to residents’ park consumption demands (5 periods).

Type	Variables	Annual growth (%)	Elastic coefficient (%)	*p*-Value	Contribution (%)	Whole contribution (%)
Natural landscape construction	Per capita area	5.75	0.345	a	49.72	61.52
	Spatial distribution	2.05	0.102	b	5.24	
	Landscape diversity	2.94	0.089	c	6.56	
Infrastructure construction	Footpaths	137.82	0.004	c	13.82	23.68
	Fitness facilities	24.15	0.009	c	5.45	
	Public chairs (stools)	36.67	0.004	c	3.68	
	Toilets	14.79	0.002	d	0.74	
Management and maintenance construction	Average opening time	1.39	0.19	b	6.62	9.53
	Parks without walls	11.36	0.004	c	1.14	
	Personnel with professional qualifications	8.85	0.008	a	1.77	
Public transportation construction	Visitors taking public transportation	23.31	0.009	c	5.26	5.26

**Note:**

a: p< 0.01

b: p< 0.05

c: p< 0.1and

d: p< 0.15.

The construction of urban public parks in Beijing has the following characteristics.

(1). Natural landscape construction. The contribution of per capita area of urban public parks is relatively enormous to residents’ utilization of urban public parks. The contribution of spatial distribution and diversity are much smaller compared to that of per capita area.

(2). Infrastructure construction. The construction of footpaths and fitness facilities have relatively higher contribution rates, while road chair (stool) construction has a lower contribution rate, and public toilet construction has the lowest contribution rate although they facilitate public travel. The public toilets have the least contributions possibly due to in-adequate toilets around the streets and pocket parks.

(3). The management and maintenance. In the case of safety and stability, the management and maintenance of urban public parks are gradually developing to improve soft power which is defined by openness and service quality of urban public parks. The average service time of urban public parks in Beijing has been extended with more parks opening all day or extending opening time, despite its slowest growth speed. The extension of opening time greatly increases the space-time capacity of urban public parks and contributes the most for this kind of construction. The proportion of parks without walls has the fastest annual growth, though of the least importance in this kind of construction. Institutions managing parks pay more attention to the employee with professional qualifications but the contribution of personnel is not as large as expected.

(4). Public transportation construction. The improvement of urban public park service quality also relies on the cooperation and assistance of external environments. Transportation is a kind of external construction and contributes greatly to the use of the service function of urban public parks. It provides convenience for residents who live far away to visit the park, facilitates their daily ecosystem service consumption activities, extends the daily service radius, and opens the full radiation of urban public parks, which releases and expands the services of urban public parks. The constructions of urban public parks should create a service pattern that relies on both internal and external environments in the future.

#### 3.2.3. Contribution trends of constructions to the residents’ park consumption demands (5 periods) (model 3)

The total contributions made by the management and maintenance, and the transportation are much lower than expected. This may be explained by the model which is based on the data of 26 years (1993–2008). Contributions of these two constructions would be larger with time going, which can be proved by dividing the time into smaller periods.

**Fig 3:**
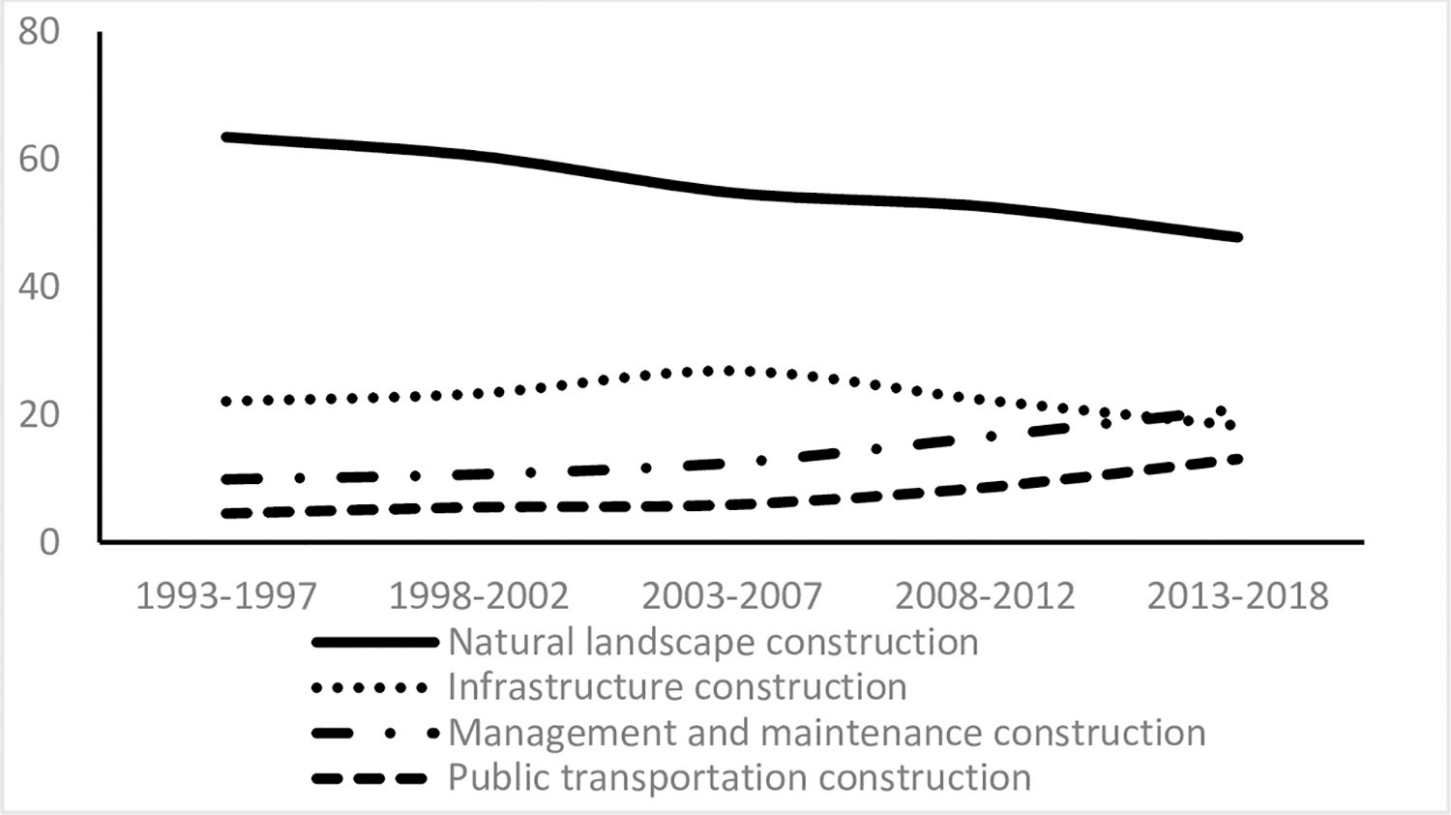
Trend analysis of the contributions made by different constructions in the past 26 years.

The full dataset was divided into five smaller datasets and each data set constructed a panel data respectively, where the number is 12 and the period is 5 or 6. The measurement results are shown in Table 9 and plotted in Fig 3.

**Table 9:** Trend analysis of the contribution of different constructions to residents’ park consumption demands.

Period	Natural landscape construction (%)	Infrastructure construction (%)	Management and maintenance construction (%)	Public transportation construction (%)
1993–1997	63.5	22.1	9.87	4.53
1998–2002	60.5	23.32	10.67	5.51
2003–2007	54.78	26.87	12.45	5.9
2008–2012	52.6	22.3	16.52	8.5
2013–2018	47.79	18.27	20.89	13.05

The contribution of the natural landscape construction of urban public parks in Beijing has been in decline since 1993, although it is the highest at present. The contribution of infrastructure first increased and then declined, with the turning point occurring in 2005. The management and maintenance and public transportation contributed the least, though their contributions were once on an upward trend, with the former rising more rapidly.

## 4. Discussion

Compared with the existing literature, this study features the following three aspects. First, in terms of the research method, this paper uses the production function and panel model to conduct a multi-period dynamic analysis of the daily ecosystem service consumption contribution rate, and the analysis results are more credible, while the existing literature used Probit and Logistics models to analyze in a certain period of time. Second, in terms of data acquisition, the existing research used statistical data or survey data, while our study uses statistical data and survey data to enrich the data. Third, in terms of the conclusion, the existing literature centered on the contribution of a single factor, while our paper is more comprehensive by reviewing, classifying and analyzing all the factors that may contribute to the daily ecosystem service consumption.

This paper reveals the dynamic relationship between the supply and demand for daily ecosystem service consumption, and provides practical and feasible solutions to better meet residents’ daily ecosystem service consumption needs. There are some other views to research this topic. First, issues from the individual micro perspective can be explored by including more comprehensive micro variables, such as income, leisure time, and family support burden. Second, the effect of daily park visiting on physical and mental health can be carried out which can be used to enhance physical health care and mental health of visitors. In the future, the data acquisition can make full use of the big data crawler technology to obtain more accurate and richer data, thus deepening the relevant research.

## 5. Conclusions and implications

### 5.1. Conclusions

Contributions of urban public park constructions will generally go through the internal (constructions of natural landscape and infrastructure) to the external development (construction of management and maintenance, and surrounding transportation) process, all of which can better satisfy the residents’ demands for daily ecosystem service consumptions. Research finds that the demands, such as frequency, duration, participation rate, and evaluation of people’s daily ecosystem service consumption have been in increasing in the past 25 years. The contributions made by internal constructions have been in decline since 1993, though they were still the greatest until 2018. The contributions made by external constructions around urban public parks have been rising, with the significant points occurring after the 2008 Olympic Games.

### 5.2. Managerial implications

Urban public park should be constructed from "hard" to the "soft" to satisfy residents ecosystem services demands. The primary construction of parks is to expand area and improve infrastructure. Therefore, in areas where the construction of the park is relatively backward, it is important to expand the scale, optimize the spatial distribution, enrich the landscape structure, and improve the construction of related fitness facilities to meet demands of ecosystem services consumption. If the basic construction work is relatively completed, more attention should be paid to enhancing the professions of management and maintenance and transportation which will improve the comprehensive strength of urban public park to provide services to residents. Besides, park construction should also pay attention to the external construction to facilitate the daily consumption of residents. For example, public transports around parks provide convenience for visitors and facilitate the daily ecosystem service consumption of residents living far away which will extend the service radius and open up the service functions of parks.

In conclusion, urban public parks firstly need to expand per capita area, optimize spatial distributions, enrich the diversity of landscape, and improve the fitness facilities to enhance the inherent quality of ecosystem services. To lay the foundation for fully releasing the service functions, urban public park constructions should also attach importance to optimizing the management and maintenance, and transportation of parks.

## Supporting information

S1 File (DOCX)

S1 Dataset (XLSX)
