# Supplementary material for: Contributions of park constructions to residents’ demands of ecosystem services consumption: A case study of urban public parks in Beijing
Source: PLoS One. 2021 Dec 15;16(12):e0259661. doi: 10.1371/journal.pone.0259661 (PMC8673618; doi:10.1371/journal.pone.0259661)
Supplement: S1 File — (DOCX) [file pone.0259661.s001.docx]

Appendix ：Questionnaires

Appendix 1 Infrastructure construction

| Workplace：XXXXX District Landscaping Bureau | | | Department: XXX | | Date: | | |
| --- | --- | --- | --- | --- | --- | --- | --- |
| Mail： | | | Tel： | | | | |
| Year | Number of footpaths | Length of footpaths（km） | Number of fitness facilities | Number of public chairs (stools) | Number of toilets | Average opening hours | Number of visitors |
| 1993 |  |  |  |  |  |  |  |
| 1997 |  |  |  |  |  |  |  |
| 2001 |  |  |  |  |  |  |  |
| 2006 |  |  |  |  |  |  |  |
| 2007 |  |  |  |  |  |  |  |
| 2008 |  |  |  |  |  |  |  |
| 2009 |  |  |  |  |  |  |  |
| 2010 |  |  |  |  |  |  |  |
| 2011 |  |  |  |  |  |  |  |
| 2012 |  |  |  |  |  |  |  |
| 2013 |  |  |  |  |  |  |  |
| 2014 |  |  |  |  |  |  |  |
| 2015 |  |  |  |  |  |  |  |
| 2016 |  |  |  |  |  |  |  |
| 2017 |  |  |  |  |  |  |  |
| 2018 |  |  |  |  |  |  |  |

Appendix 2 Maintenance company interview (Management and maintenance construction)

| Workplace: | | | | Name： | Position： | | |
| --- | --- | --- | --- | --- | --- | --- | --- |
| Tel： | | | | | | | |
| Year | Average stay time of visitors (min) in summer | Average stay time of visitors (min) in winter | Proportion of days exceeding the approved carrying capacity of the park | Personnel with professional qualifications（%） | Parks without walls（%） | 24-hour parks (by area, %) | Non-24-hour park |
| 1993 |  |  |  |  |  |  |  |
| 1998 |  |  |  |  |  |  |  |
| 2003 |  |  |  |  |  |  |  |
| 2008 |  |  |  |  |  |  |  |
| 2013 |  |  |  |  |  |  |  |
| 2018 |  |  |  |  |  |  |  |

Appendix 3 Visitors interview (Public transport construction)

| Interview place： | | | | Number of interviewees： | | Date： |
| --- | --- | --- | --- | --- | --- | --- |
| Average age： | | | | | | |
| Year | Average stay time of visitors (min, summer, construction level) | Average stay time of visitors (min, winter, construction level) | Distance to park (km) | Walking or cycling, % | Public transportation, % | Other transportation |
| 1993 |  |  |  |  |  |  |
| 1998 |  |  |  |  |  |  |
| 2003 |  |  |  |  |  |  |
| 2008 |  |  |  |  |  |  |
| 2013 |  |  |  |  |  |  |
| 2018 |  |  |  |  |  |  |

Appendix 4 Visitors interview (Visiting motivation)

1、Address：____________

2、Gender: ___________

3、Age：_______

4、Please fill in the following form according to your memory. If the corresponding year you are not born or the memory is not clear, you do not need to fill in.

| Year | Motivation（choose two main motivations） | Average visit times per week | Score of crowding in park (1-100) | Score of ecological landscape of Park (1-100) |
| --- | --- | --- | --- | --- |
| 25 years ago (1993, if not born or not clearly, needn't fill in) | A. Commercial entertainment; B. Free entertainment facilities; C. Animals and plants; D. High-quality ecosystem services; E. *Others | A.1; B.2; C.3; D.4; E.5; F. Others |  |  |
| 20 years ago (1998, if not born or not clearly, needn’t fill in) | A. Commercial entertainment; B. Free entertainment facilities; C. Animals and plants; D. High-quality ecosystem services; E. *Others | A.1; B.2; C.3; D.4; E.5; F. Others |  |  |
| 15 years ago (2003, if not born or not clearly, needn't fill in) | A. Commercial entertainment; B. Free entertainment facilities; C. Animals and plants; D. High-quality ecosystem services; E. *Others | A.1; B.2; C.3; D.4; E.5; F. Others |  |  |
| 10 years ago (2008, if not born or not clearly, needn't fill in) | A. Commercial entertainment; B. Free entertainment facilities; C. Animals and plants; D. High-quality ecosystem services; E. *Others | A.1; B.2; C.3; D.4; E.5; F. Others |  |  |
| 5 years ago (2013, if not born or not clearly, needn't fill in) | A. Commercial entertainment; B. Free entertainment facilities; C. Animals and plants; D. High-quality ecosystem services; E. *Others | A.1; B.2; C.3; D.4; E.5; F. Others |  |  |
| Present | A. Commercial entertainment; B. Free entertainment facilities; C. Animals and plants; D. High-quality ecosystem services; E. *Others | A.1; B.2; C.3; D.4; E.5; F. Others |  |  |
